# The complete chloroplast genome of *Pleione pleionoides* (Orchidaceae)

**DOI:** 10.1080/23802359.2019.1624215

**Published:** 2019-07-10

**Authors:** Jin-Liao Chen, Zhong-Wu Dai, Xiao-Yu Dai, Si-Ren Lan, Sha-Sha Wu

**Affiliations:** a Key Laboratory of National Forestry and Grassland Administration for Orchid Conservation and Utilization at College of Landscape Architecture, Fujian Agriculture and Forestry University, Fuzhou, China;; b Logistics Management Department, Fujian Agriculture and Forestry University, Fuzhou, China;; c Fujian Ornamental Plant Germplasm Resources Innovation and Engineering Application Research Center, Fuzhou, China

**Keywords:** Chloroplast genome, phylogenetic, Illumina sequencing, *Pleione pleionoides*

## Abstract

*Pleione pleionoides* is a vulnerable orchid with significant ornamental values. Here, we report the first complete chloroplast genome of *P. pleionoides*. The circular genome was 159,468 bp in length and consisted of a pair of inverted repeats (IR 26,651 bp), which were separated by a large single copy region (LSC 87,461 bp) and a small single copy region (SSC 18,705 bp). It contained 115 unique genes, including 87 protein-coding genes, 38 tRNAs, and 8 rRNAs. The maximum likelihood phylogenetic analysis indicated that *P. pleionoides* and *P. bulbocodioides* cluster together and closely related to *P. formosana*.

Braem and Mohr (1989) is a terrestrial orchid, which grows on humus-covered or mossy rocks, cliffs in forests, between 1700–2300 m in Chongqing, Guizhou, and Western Hubei province of China (Chen et al. 2009). It is a vulnerable species in the Red List (IUCN 2018) and the most strikingly attractive species in the *P. bulbocodioides* complex (Cribb and Butterfield 1999), which has been thought to be the source of persistent confusion and ambiguity in taxonomy of *Pleione* (Gravendeel et al. 2004). So far, the complete chloroplast (cp) genome about *P. bulbocodioides* complex such as *P. bulbocodioides* (Shi et al. 2018) and *P. formosana* (Jiang et al. 2019) have been reported. In this study, we aimed to assemble and characterize *P. pleionoides* cp genomes. These data will be useful for phylogenetic studies and provide a better understanding on the evolution of genera *Pleione*.

Samples of *P. pleionoides* were collected from Taibai, Shanxi province of China. Total genomic DNA was extracted from leaf tissue (Voucher specimen: 34°2′1.63″N 107°52′11.06″E, TB3-2, FAFU) following the protocol described by Doyle and Doyle (1987). After the construction of shotgun library, high-throughput sequencing was conducted on the Illumina Hiseq 2000 sequencing platform. Raw reads were filtered by NGS QC Toolkit (Patel and Jain 2012). The clean reads were firstly aligned to *P. bulbocodioides* (Genbank Accession No. KY849819) and *P. formosana* (Genbank Accession No. MK361027). Filtered reads were then assembled into contigs in the software Platanus version 1.2.4 (Kajitani et al. 2014). After it is assembled, the obtained scaffolds and contigs were assembled into cp genome by Geneious version 11.1.5 (Kearse et al. 2012), using the algorithm MUMmer. The genome was automatically annotated using DOGMA (Wyman et al. 2004), then adjusted by Geneious version 11.1.5 (Kearse et al. 2012). The validated complete cp genome sequence was submitted to GenBank with accession number MK810725. The length of *P. pleionoides* cp genome sequence is 159,468 bp, containing a large single copy (LSC) region of 87,461 bp and a small single copy (SSC) region of 18,705 bp, and two inverted repeat (IR) regions of 26,651 bp. The cp genome encoded 135 genes, of which 113 were unique genes (87 protein-coding genes, 38 tRNAs, and 8 rRNAs). Overall, GC content of the whole genome is 37.2%, while the corresponding values of the LSC, SSC, and IR regions are 35.0, 30.4, and 43.4%, respectively.

Phylogenetic analysis was conducted to investigate taxonomic positions of *P. pleionoides* within Epidendroideae. Eighty-five complete cp genomes of Epidendroideae and two species of Orchidoideae were aligned using HomBlocks pipeline (Bi et al. 2017). RAxML-HPC Black-Box version 8.1.24 (Stamatakis et al. 2008) was used to construct a maximum likelihood tree with *Ludisia discolor* and *Goodyera fumata* as an outgroup. The branch support was computed with 1000 bootstrap replicates. The ML tree analysis indicated that *P. pleionoides* and *P. bulbocodioides* cluster together and closely related to *P. formosana* with 100% bootstrap support (Figure 1).

**Figure 1.**
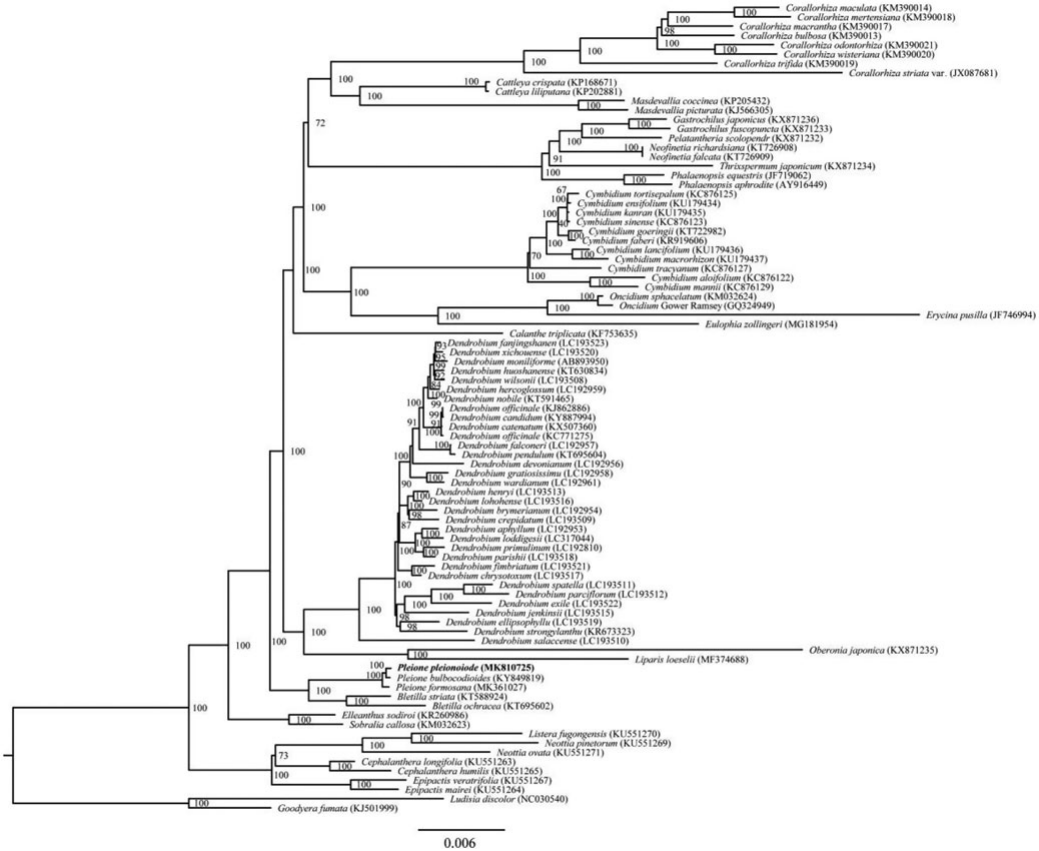
Maximum-likelihood (ML) tree based on 85 complete cp genome of Epidendroideae, with *Goodyera fumata* and *Ludisia discolor* (Orchidoideae) as an outgroup. The bootstrap value indicated on each node and the position of *Pleione pleionoides* is shown in bold.
